# Comparison of Short and Continuous Hydration Regimen in Chemotherapy Containing Intermediate- to High-Dose Cisplatin

**DOI:** 10.1155/2014/767652

**Published:** 2014-09-24

**Authors:** Akira Ouchi, Masahiko Asano, Keiya Aono, Tetsuya Watanabe, Takehiro Kato

**Affiliations:** Department of Surgery, Chita City Hospital, 2-1 Nagai, Shinchi, Chita, Aichi 478-0017, Japan

## Abstract

*Aim.* The efficacy of the short hydration regimen was reported in chemotherapy containing intermediate- to high-dose cisplatin, and the use of outpatient chemotherapy containing cisplatin with short hydration has been widespread in recent years. *Methods.* We compared patients with gastric cancer, lung cancer, and urothelial cancer who received outpatient chemotherapy containing cisplatin (≥60 mg/m^2^/cycle) with the short hydration regimen since April 2012 (*n* = 13) with those who received hospital chemotherapy with continuous hydration between April 2011 and March 2013 (*n* = 17) in our hospital. *Results.* Grade 2 or higher acute kidney injury occurred in 2 patients in the continuous hydration group and in no patient in the short hydration group; 1 patient discontinued treatment on account of nephrotoxicity. There was no difference between the 2 groups in maximum creatinine increment and maximum clearance decrement. Relative dose intensity in the short hydration group was higher than that in the continuous hydration group (89.5% versus 80.3%; *P* < 0.01). *Conclusions.* The short hydration regimen in outpatient chemotherapy containing intermediate- to high-dose cisplatin is as safe as the continuous hydration regimen and increased the efficacy of chemotherapy.

## 1. Introduction

Cisplatin is a most frequently and widely used anticancer drug for the treatment of solid cancers including gastric cancer, urothelial cancer, and lung cancer. Cisplatin exhibits strong anticancer efficacy although it is associated with several toxicities including nausea, neuropathy, ototoxic effects, myelosuppression, and nephrotoxicity. Nephrotoxicity is the major dose-limiting effect of cisplatin and it sometimes leads to cessation of chemotherapy [1, 2]. Nephrotoxicity is reduced but not completely prevented by different measures such as dose fractionation, slower infusion rate, forced diuresis with diuretics, and hydration [3–6]. Continuous hydration for over 24 h is recommended after cisplatin administration to reduce nephrotoxicity, but it requires admission to hospital for chemotherapy. Chemotherapy in hospital hinders the daily life of patients receiving chemotherapy and is complicated for medical staff. Moreover, hospital treatment costs add up quickly. Consequently, continuous hydration decreases the dose intensity and the treatment efficacy because of the complexity of chemotherapy regimen.

In 2007, Tiseo et al. reported the efficacy of a short hydration regimen for chemotherapy patients receiving intermediate- to high-dose cisplatin [7]. The short hydration regimen is recommended for only a few hours after cisplatin administration and does not require admission to hospital for chemotherapy. Outpatient chemotherapy is convenient for patients receiving chemotherapy and simple for medical staff. Consequently, short hydration increases the dose intensity and the treatment efficacy because of the simplicity of chemotherapy regimen. Furthermore, several antiemetic drugs including NK1 receptor antagonist and 5-HT3 receptor antagonist are used in combination with chemotherapy containing cisplatin, thus making the management of adverse events including nausea and anorexia in outpatient departments easier.

Outpatient chemotherapy containing cisplatin with short hydration has increased in use in recent years. However, only a few studies have evaluated the clinical outcome of chemotherapy containing cisplatin with short hydration, particularly its noninferiority to continuous hydration [8, 9]. The aim of this study was to evaluate the safety and efficacy of short hydration to retrospectively compare the clinical outcome of chemotherapy in hospital with continuous hydration and outpatient chemotherapy with short hydration.

## 2. Subjects and Methods

### 2.1. Subjects

Between January 2011 and December 2013, we retrospectively evaluated 30 patients with gastric cancer, lung cancer, and urothelial cancer who received chemotherapy containing intermediate- to high-dose cisplatin (≥60 mg/m^2^/cycle) in our hospital. We started outpatient administration of cisplatin with short hydration in July 2012 and divided the patients into two groups based on this period: the continuous hydration group (CH group, before June 2012, 17 patients) and the short hydration group (SH group, after July 2012, 13 patients). The clinical variables of each patient were retrospectively examined on the basis of medical records of sex, age, height, weight, type of disease, combined anticancer drugs, and total number of cycles. Body surface area (BSA) was calculated from the height and weight of the patients by the DuBois and DuBois formula [10].

### 2.2. Treatment

Patients in the CH group received either cisplatin-based chemotherapy with continuous hydration with normal saline or maintenance solution for over 24 h in hospital at each cycle. Patients in the SH group received cisplatin-based chemotherapy with our short hydration regimen, which was based on the regimen reported by Tiseo et al. [7]. Cisplatin was dissolved in 500 mL of normal saline and infused with 2000 mL of normal saline and administrated over 4 h with 20 mg of i.v. furosemide (Table 1). Cisplatin dose for each patient was recorded at each cycle. Relative dose intensity was calculated from BSA and cisplatin dose. To evaluate cisplatin nephrotoxicity, serum creatinine levels were measured before every chemotherapy cycle and as necessary. Creatinine clearance values were calculated from sex, age, weight, and serum creatinine levels by the Cockcroft-Gault formula [11]. To compare cisplatin nephrotoxicity between the CH and SH groups, the prechemotherapy serum creatinine levels (*C*_pre_), maximum serum creatinine levels during the treatment (*C*_max⁡_), and their clearance values (*Cl*_pre_ and *Cl*_min⁡_, resp.) were examined in each patient. The maximum creatinine increment (*I*_max⁡_ = *C*_max⁡_ − *C*_pre_) and the corresponding maximum clearance decrement (*D*_max⁡_ = *Cl*_pre_ − *Cl*_min⁡_) were also calculated.

### 2.3. Statistical Analyses

The means and standard deviations (SD) of the collected data were calculated. The Mann-Whitney *U*-test was used for statistical analyses, and *P* values of <0.05 were considered significant. JMP software version 5.0.1 (SAS institute Inc., Cary, NC, USA) was used for all statistical analyses.

## 3. Results

### 3.1. Clinical Demographics

There were no differences in sex, age, height, weight, and BSA between the 2 groups (Table 2). In the 17 patients of the CH group, 14 had gastric cancer, two had urothelial cancer, and one had lung cancer. In the 13 patients of the SH group, five had gastric cancer, three had urothelial cancer, and three had lung cancer. In the CH group, cisplatin was combined with titanium silicate- (TS-) 1 in 14 patients, with gemcitabine in two and with etoposide in one. In the SH group, cisplatin was combined with TS-1 in five patients, with gemcitabine in two, with capecitabine in one, with irinotecan in one, and with etoposide in one. There were no differences in serum creatinine levels and creatinine clearance values before chemotherapy between the two groups.

### 3.2. Treatment Outcomes

The mean number of cycles was 2.6 ± 1.3 and 3.6 ± 2.5 in the CH and SH groups, respectively (Table 3). The mean cisplatin-planned dose intensity was 13.4 ± 3.1 mg/m^2^/week and 13.9 ± 4.4 mg/m^2^/week in the CH and SH groups, respectively. There were no differences in the mean cisplatin-planned dose intensity between the two groups (*P* = 0.42 and 0.71, resp.). The mean relative dose intensity was 80.3% ± 9.3% and 89.5% ± 6.5% in the CH and SH groups, respectively. Relative dose intensity was significantly higher (*P* < 0.01) in the SH group than that in the CH group. Grade 2 or higher acute kidney injury (according to Common Terminology Criteria for Adverse Events of National Cancer Institute) occurred only in two patients in the CH group (Table 4). Grade 2 or higher creatinine increases occurred in three patients and one patient in the CH and SH groups, respectively. One patient in the CH group discontinued treatment on account of nephrotoxicity after the 2nd cycle of cisplatin administration, whereas no patient in the SH group discontinued treatment on account of nephrotoxicity. The mean *I*_max⁡_ was 0.32 ± 0.57 mg/dL and 0.14 ± 0.15 mg/dL in the CH and SH groups, respectively. The mean *D*_max⁡_ was 18.3 ± 20.2 mg/min and 10.9 ± 13.3 mg/min in the CH and SH groups, respectively. There was no difference in *I*_max⁡_ and *D*_max⁡_ (*P* = 0.43 and 0.28, resp.) between the two groups.

## 4. Discussions

Cisplatin nephrotoxicity is reduced by the use of diuretics and hydration, which reduces cisplatin concentration and the contact time with tubular epithelium [3–6], but it is not completely prevented. Hydration with normal saline is recommended for preventing cisplatin nephrotoxicity, but there is no clear data on the appropriate amount and duration of hydration. Furthermore, there is no consensus on a standard cisplatin hydration regimen. The instructions of most commercially available cisplatin formulations recommended continuous hydration for over 24 h. Based on these recommendations, a continuous hydration regimen in hospital for over 24 h is currently used to reduce cisplatin nephrotoxicity in many institutions worldwide [12, 13].

Immediately and irreversibly, cisplatin binds to plasma protein after i.v. injection, and free platinum derived from cisplatin, which is the cause of nephrotoxicity, radically decreases within a few hours [14]. Free platinum derived from cisplatin is reduced to one-tenth of its original concentration within the first 2 h after the end of cisplatin infusion and is under the measurement limit within the next 2 h. This fact leads to the presumption that a few hours are sufficient for hydration after cisplatin administration to reduce the concentration of free platinum derived from cisplatin and the contact time with tubular epithelium. In addition, Stewart et al. reported that hydration of over 2000 mL of normal saline did not reduce nephrotoxicity [15]; that is, hydration for a few hours and with 2000 mL of normal saline is recommended after cisplatin administration to reduce nephrotoxicity. Based on the above results, in 2007, Tiseo et al. advocated the “short hydration regimen” of intermediate- to high-dose cisplatin-based chemotherapy for outpatient treatment in lung cancer and mesothelioma and reported the feasibility [7].

The present study retrospectively evaluated the safety and efficacy of short hydration to compare the clinical outcome of chemotherapy in hospital with continuous hydration and outpatient chemotherapy with short hydration. In the present study, chemotherapy interruption caused by renal toxicity occurred in only 3.3% of patients (one of 30 patients), which is similar to that previously reported [16]. There was no difference between the two groups in *I*_max⁡_ and *D*_max⁡_. Furthermore, acute kidney injury and creatinine increases in the SH group were equal to or less than those in the CH group. These results suggest that the safety of short hydration is equivalent to that of the continuous hydration in terms of nephrotoxicity.

Regarding the efficacy of short hydration in the present study, although there were no differences between the two groups in mean cisplatin-planned dose intensity, relative dose intensity was significantly higher in the SH group than in the CH group. A continuous hydration regimen requires admission to hospital for patients. Chemotherapy in hospital hinders the daily life of patients receiving chemotherapy. In contrast, a short hydration regimen does not require admission to hospital. Outpatient chemotherapy does not hinder the daily life. The convenience of short hydration regimen improved compliance with treatment, increased relative dose intensity, and eventually increased the efficacy of chemotherapy containing cisplatin.

The short hydration regimen has cost effective advantages. The short hydration regimen is able to reduce costs and resource utilization without any loss on the quality of patient care. A Memorial Sloan-Kettering Cancer Center study reported that day hospital care of oncology patients is clinically equivalent to that of in-patient care, causes no negative psychosocial effects, and costs less than in-patient care [17]. It is beneficial to reduce treatment costs not only for patients receiving chemotherapy but also on medical equipment for hospitals.

Our study has several limitations that need to be addressed in future research. First, mean cisplatin-planned dose intensity in the present study is lower than that previously reported. Cisplatin is often used at 60–80 mg/m^2^/cycle for 3–5 weeks in Japan, and this dose is slightly less profitable than Euro-American doses. Second, in the present study, cisplatin was combined with various other anticancer drugs in all 30 cases. It is possible that not only cisplatin but also other anticancer drugs affected renal function during the course of treatment. Third, this study was a retrospective analysis of a small number of patients. A randomized control study with large patient numbers using a uniform protocol is required to significantly reveal the safety and efficacy of the short hydration regimen.

## 5. Conclusions

The short hydration regimen in outpatient chemotherapy containing intermediate- to high-dose cisplatin is as safe as the continuous hydration regimen and increased the efficacy of chemotherapy.

## Figures and Tables

**Table 1:** Our short hydration regimen.

Fluids	Drugs	Timing
100 mL of 0.9% NaCl_aq_	Dexamethasone (12 mg) + 5-HT3 antagonist	15 min
1000 mL of 0.9% NaCl_aq_	MgSO_4_ (8 mEq)	120 min
500 mL of 0.9% NaCl_aq_	Cisplatin (60–80 mg/m^2^)	60 min
	Furosemide (20 mg)	Bolus
500 mL of 0.9% NaCl_aq_		60 min

NaCl_aq_: sodium chloride solution.

MgSO_4_: magnesium sulfate.

**Table 2:** Demographics of the two groups.

	CH (*n* = 17)	SH (*n* = 13)	*P* value
Sex (male : female)	11 : 6	10 : 3	0.46
Age (years)	67.1 ± 7.0	71.1 ± 7.3	0.14
Height (cm)	160.0 ± 8.5	160.0 ± 7.6	0.93
Weight (kg)	51.5 ± 8.5	56.2 ± 10.7	0.20
BSA (m^2^)	1.51 ± 0.15	1.57 ± 0.17	0.43
Type of disease			
Gastric cancer	14	5	
Urothelial cancer	2	5	
Lung cancer	1	3	
Combined with			
TS-1	14	5	
Gemcitabine	2	5	
Capecitabine		1	
Irinotecan		1	
Etoposide	1	1	
Cr (mg/dL)	0.71 ± 0.19	0.83 ± 0.27	0.32
CCr (mL/min)	73.2 ± 19.3	68.5 ± 25.1	0.39

Values are mean ± SD.

CH: continuous hydration.

SH: short hydration.

BSA: body surface area.

Cr: creatinine.

CCr: creatinine clearance.

**Table 3:** Results of cisplatin administration.

	CH (*n* = 17)	SH (*n* = 13)	*P* value
Number of cycles	2.6 ± 1.3	3.6 ± 2.5	0.42
PDI (mg/m^2^/week)	13.4 ± 3.1	13.9 ± 4.4	0.71
RDI (%)	80.3 ± 9.3	89.5 ± 6.5	0.01∗

Values are mean ± S.D.

CH: continuous hydration.

SH: short hydration.

PDI: planned dose intensity.

RDI: relative dose intensity.

*P value < 0.05.

**Table 4:** Nephrotoxicity after cisplatin administration.

	CH (*n* = 17)	SH (*n* = 13)	*P* value
AKI			
Grade 1	1	2	
Grade 2	0	0	
>Grade 3	2^†^	0	
Creatinine increase			
Grade 1	12	9	
Grade 2	1	1	
>Grade 3	2	0	
*I*_max⁡_ (mg/dL)	0.32 ± 0.57	0.14 ± 0.15	0.43
*D*_max⁡_ (mg/min)	18.3 ± 20.2	10.9 ± 13.3	0.28

Values are mean ± SD.

CH: continuous hydration.

SH: short hydration.

AKI: acute kidney injury.

*I*_max⁡_: maximum creatinine increment.

*D*_max⁡_: maximum clearance decrement.

^†^One patient discontinued treatment on account of nephrotoxicity.
